# Multimodal integration in statistical learning: evidence from the McGurk illusion

**DOI:** 10.3389/fpsyg.2014.00407

**Published:** 2014-05-16

**Authors:** Aaron D. Mitchel, Morten H. Christiansen, Daniel J. Weiss

**Affiliations:** ^1^Department of Psychology and Program in Neuroscience, Bucknell UniversityLewisburg, PA, USA; ^2^Department of Psychology, Cornell UniversityIthaca, NY, USA; ^3^Department of Language and Communication, University of Southern DenmarkOdense, Denmark; ^4^Haskins Laboratories, New HavenCT, USA; ^5^Department of Psychology and Program in Linguistics, Pennsylvania State University, University ParkPA, USA

**Keywords:** multisensory statistical learning, statistical learning mechanisms, multisensory perception, language acquisition, McGurk illusion, multisensory integration, audiovisual speech perception

## Abstract

Recent advances in the field of statistical learning have established that learners are able to track regularities of multimodal stimuli, yet it is unknown whether the statistical computations are performed on integrated representations or on separate, unimodal representations. In the present study, we investigated the ability of adults to integrate audio and visual input during statistical learning. We presented learners with a speech stream synchronized with a video of a speaker’s face. In the critical condition, the visual (e.g., /gi/) and auditory (e.g., /mi/) signals were occasionally incongruent, which we predicted would produce the McGurk illusion, resulting in the perception of an audiovisual syllable (e.g., /ni/). In this way, we used the McGurk illusion to manipulate the underlying statistical structure of the speech streams, such that perception of these illusory syllables facilitated participants’ ability to segment the speech stream. Our results therefore demonstrate that participants can integrate audio and visual input to perceive the McGurk illusion during statistical learning. We interpret our findings as support for modality-interactive accounts of statistical learning.

## INTRODUCTION

Over the last 15 years, a growing body of research has detailed language learners’ ability to extract statistical regularities from speech (hereafter *statistical learning*), particularly in the domain of speech segmentation. Many studies of statistical learning have examined this ability in the context of a single input modality, including auditory (Saffran et al., 1996, 1999), visual (Fiser and Aslin, 2002), and tactile stimuli (Conway and Christiansen, 2005). However, since the learning environment is typically multimodal (Stein and Stanford, 2008), perceptual mechanisms may be tuned to operate optimally over multimodal input, suggesting that unimodal indices of perceptual learning could underestimate their capacity (Shams and Seitz, 2008). Consequently, there has been a recent increase in research investigating how statistical learning mechanisms track multimodal input (e.g., Sell and Kaschak, 2009; Cunillera et al., 2010; Mitchel and Weiss, 2010, 2013; Thiessen, 2010). Numerous studies have demonstrated that adults are capable of successfully tracking multiple statistical inputs simultaneously in separate modalities (Conway and Christiansen, 2006; Seitz et al., 2007; Emberson et al., 2011; Mitchel and Weiss, 2011), though the underlying processes remain unclear. When learning from multimodal input, do learners develop independent unimodal representations, a single multimodal representation, or some combination of the two? In the present study, we investigate this issue by exploring the influence of the McGurk illusion (a well-attested demonstration of audiovisual integration; McGurk and MacDonald, 1976) on multimodal statistical learning.

In one of the initial studies on multimodal statistical learning, Seitz et al. (2007) simultaneously presented participants with an audio stream (non-tonal noises) and a visual stream (2-D shapes). At test, participants were able to correctly identify statistically defined audio, visual, and audiovisual bigrams that had appeared in the familiarization stream, demonstrating that learners are able to extract multiple, concurrent statistical patterns across sensory modalities. Moreover, Seitz et al. did not observe disparities in performance when the streams were presented together or in isolation. Therefore, the authors concluded that statistical learning in one modality is processed independently from input in another modality. In contrast, a more recent study has provided evidence of cross-modal effects during multimodal statistical learning that are inconsistent with modality-independence (Mitchel and Weiss, 2011). In this study, adult learners were able to segment visual and auditory (tone) sequences simultaneously when triplet boundaries across streams were in-phase, replicating the findings of Seitz and colleagues. However, learning was disrupted when the streams were offset such that the triplet boundaries across modalities were misaligned. This decrement in performance suggests that statistical learning of multimodal inputs are subject to cross-modal interference, as the relationship of boundary information between streams influenced participants’ ability to segment each stream (Mitchel and Weiss, 2011). We proposed that statistical learning may be governed by an interactive network of modality-specific mechanisms. In this view, learning is constrained by the modality of the input (see Conway and Christiansen, 2005, 2006) while cross-modal effects operate via associative links between mechanisms (Mitchel and Weiss, 2010, 2011; Emberson et al., 2011; Glicksohn and Cohen, 2013; see also Cunillera et al., 2010).

While the aforementioned studies provide evidence that statistical learning mechanisms are capable of processing multimodal input, what is encoded from this input remains unclear. Specifically, when information from multiple modalities is available, are statistical computations performed on integrated, multimodal percepts or on unimodal representations? Although multimodal integration, or the coupling of two or more senses to produce a coherent multimodal representation, is a central property of perception (Shimojo and Shams, 2001), no study, to the best of our knowledge, has investigated this process in the context of statistical learning. A goal of the present study, then, is to investigate multimodal integration in statistical learning; specifically, we utilize the McGurk illusion to examine whether statistical learning of speech input operates on auditory input alone or on an integrated audiovisual representation.

The McGurk illusion (McGurk and MacDonald, 1976) arises when incongruous visual information (e.g., lip movements) alters the auditory perception of speech. For example, one form of the McGurk illusion occurs when synchronously presented incongruent audio (e.g., /ba/) and visual (e.g., /ga/) syllables are integrated to be perceived as *fused* syllables (e.g., /da/). The McGurk illusion is widely regarded as a compelling behavioral index of audiovisual integration (e.g., Green, 1998; Massaro, 1998; Brancazio and Miller, 2005). Here, we test how auditory statistical learning may be influenced by the perceived audiovisual syllables resulting from the McGurk illusion.

In the present study, we expose learners to a miniature artificial language that provides no transitional probability cues to word boundaries. We paired the language with a synchronous video of a speaker’s face in three conditions. In the *Audio-only* condition, the artificial speech stream is presented alone. In the *Audiovisual Consistent* condition, the speech stream is paired with a talking face display that perfectly matches the speech syllables. In the *Audiovisual Inconsistent* condition, inconsistencies between select auditory syllables and visual articulatory gestures are used to elicit a McGurk illusion that could alter the statistical structure of the artificial language. In this altered structure, the transitional probabilities should cue word boundaries, such that syllable-to-syllable transitional probabilities within words (0.50) should be greater than transitional probabilities between words (0.25). Thus, if learners compute transitional probabilities using an integrated percept, then the changes in the statistical structure of the language in the Audiovisual Inconsistent condition should enhance learning relative to the Audio-only or Audiovisual Consistent conditions.

## MATERIALS AND METHODS

### PARTICIPANTS

One hundred forty-two (98 female, 46 male) participants from Pennsylvania State University were included in the analyses. Eleven additional participants (7%) were excluded from analysis for failing to follow directions (7), such as falling asleep or removing headphones, and due to technical errors during the experiment (4).

### STIMULI

The auditory stimuli consisted of an artificial language with four tri-syllabic (CV.CV.CV) words (see **Table 1**). Six consonants and six vowels were combined to form a total of six CV syllables. Each syllable was created by synthesizing natural speech syllables and removing any acoustic cues to word boundaries in a similar manner as described in previous statistical learning experiments (see Weiss et al., 2009, 2010; Mitchel and Weiss, 2010). We recorded a male speaker producing CVC syllables, with the final consonant being one of three possible places of articulation (bilabial, alveolar, or velar). Coda consonants were recorded to preserve the co-articulatory vowel-to-consonant transitions when the CV syllables were later concatenated into trisyllabic words. Each CVC syllable was then hand-edited in Praat, removing the coda consonants and equating vowel duration. The syllables were synthesized in Praat, overlaying the same pitch (f0) contour onto each syllable in order to remove any pitch or stress cues to segmentation and then concatenated to form the words.

**Table 1:** Design of artificial language across display conditions.

	Display condition														
	Audio-only					Audiovisual consistent					Audiovisual inconsistent (McGurk)				
Words	so		bæ		ta	so		bæ		ta	so		bæ		ta
	je		lu		mi	je		lu		mi	je		lu		**ni**
	bæ		je		pa	bæ		je		pa	bæ		je		**ta**
	lu		so		ni	lu		so		ni	lu		so		ni
TPs	0.5	→	0.5	→	0.5	0.5	→	0.5	→	0.5	0.5	→	0.5	→	0.25

*Bolded syllables in the Audiovisual Inconsistent condition represent the illusory, integrated McGurk syllables. Syllable-to-syllable transitional probabilities (TPs) are reported for each condition. TPs in the audiovisual conditions reflect the statistical structures of the languages if they include the integrated percept.*

The four words were concatenated into a continuous stream in a pseudo-random order, such that each word appeared an equal number of times and no word ever followed itself. The artificial language had flat transitional probabilities within and between words (0.50 → 0.50 → 0.50; see **Table 1**). Without statistical cues to word boundary, it was predicted that this language should not be learned in the Audio-only or Audiovisual Consistent conditions. In addition, the order of words in the stream was constrained such that words 1 and 2 were only followed by words 3 and 4, and vice versa. In the Audiovisual Inconsistent condition, this order constraint allowed the McGurk illusion (if perceived) to alter the statistical structure of the entire language while only manipulating two word-final syllables. Specifically, perception of the McGurk syllables would alter the syllable inventory across which transitional probabilities were calculated. In the Audiovisual Inconsistent condition, the new, integrated syllable inventory would provide robust statistical word boundary cues (0.50 → 0.50 → 0.25; see **Table 1**); thus, it was predicted that learning should occur in the Audiovisual Inconsistent condition if participants perceived the integrated, illusory syllables. The speech stream was comprised of three 4-min blocks for a total familiarization of 12 min. Between each block there was a 1 min silence during which the screen turned white.

For the visual displays, a Sony Handicam was used to video-record an assistant lip-synching to an audio-stream while reading from a list of words mounted behind the camera (see Mitchel and Weiss, 2010, 2013). The video was then hand-edited in Adobe Premiere ^©^ to ensure that the audio stream and video display were synchronous, aligning them such that the articulatory gestures of the lips coincided with the corresponding auditory event. The video was cropped to display only the lips of the actor, and then exported as a Quicktime movie. The content of the consistent visual display was the same as the audio stream. The content of the inconsistent visual stream, however, differed from the audio stream in two word-final syllables (audio: /mi/ and /pa/, visual: /gi/ and /ka/, respectively). If these inconsistent audio and visual syllables were integrated, then participants should have perceived a McGurk illusion of /ni/ and /ta/ (MacDonald and McGurk, 1978).

Learning of the statistically defined words was tested using an audio-only, 24 item word-identification task. The same test was given for each condition and consisted of six words, three part-words, and three non-words, with each item presented twice in a randomized order. The six words were sub-divided into three classes with two words each: audiovisual, audio-only, and McGurk. Audiovisual test items were always consistent across audio and visual input during familiarization (/so bae ta/, /lu so ni/, see **Table 1**). Audio-only test items were taken from the audio stream (/je lu mi/, /bae je pa/), and should have been heard by participants if they did not perceive the McGurk illusion during familiarization. McGurk test items were the auditory equivalent of the illusory words that participants in the Audiovisual Inconsistent condition should have perceived if the McGurk illusion produced a fused, integrated percept (/je lu ni/, /bae je ta/). Non-words were combinations of syllables that did not occur together during familiarization, but conserved positional information (e.g., words with syllables ABC and DEF could form non-words AEF or DBC). Part-words were formed by combining the third syllable of one word with the first and second syllables of another word (e.g., ABC and DEF yield part-words CDE and FAB).

### PROCEDURE

Participants in all conditions provided written informed consent, and the protocol used in this experiment was approved by the Office of Research Protections at The Pennsylvania State University (IRB protocol #16986).

In the Audio-only condition, participants were instructed to listen to an audio stream and informed they would be tested on knowledge acquired from this familiarization. Participants were not informed that the audio stream was an artificial language. The familiarization stream and test were presented using E-prime software. Using E-Prime, participants were asked to judge whether the test item was a word, based on the preceding familiarization stream, by pressing the keys marked “yes” or “no” on a keyboard.

In the two audiovisual conditions, participants were instructed to view a short movie and informed that they would be tested following the movie. There were no explicit instructions given about the nature of the movie, nor were participants informed that the audio stream was composed of an artificial language. Familiarization streams were presented using iTunes (version 7.0) software. Following familiarization, participants completed the same identification test as in the audio-only condition, presented using E-Prime software. There was no video display during test.

### ANALYSIS

Using signal detection theory, *d*′ (hit rate – false alarm rate) was calculated to determine participants’ sensitivity to detecting words. Since endorsement of McGurk and Audio-only word items could be categorized as either hits or false alarms depending on the condition, we elected to define *hits* as endorsement of audiovisual-consistent word items and defined *false alarms* as endorsement of non-words (which never occurred during familiarization, providing an accurate index of false alarm rate). Thus, *d*′ was calculated by subtracting the standardized endorsement rate for non-words from the standardized endorsement rate for audiovisual words: *d*′ = *z*[*P*(“yes”| audiovisual words)] – *z*[*P*(“yes”| Non-words)]. In this task, a *d*′ of 0 represents chance performance (participants were equally likely to endorse words and non-words), while a *d*′ significantly above 0 represents learning (participants were more likely to endorse words than non-words). In order to assess the learning of the McGurk words, we compared endorsement rates (the probability that a participant would choose “yes” for an item) across display conditions.

## RESULTS

### *d*′ ANALYSIS

**FIGURE 1:**
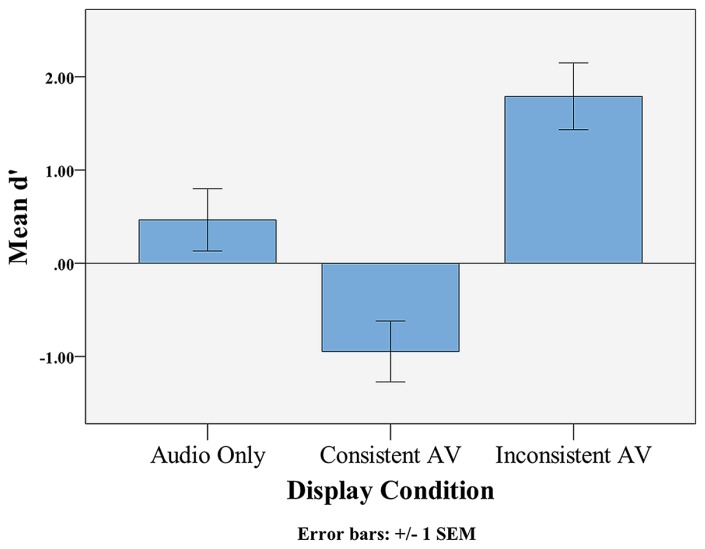
**Mean *d*′ across display types**. The horizontal line represents chance performance (*d*′ = 0).

All statistical tests were two-tailed. The mean *d*′ score in the Audio-only condition was 0.47 (SD = 2.31), a level of performance that was not significantly above chance, *t*(47) = 1.39, *p* = 0.170, Cohen’s *d* = 0.20 (see **Figure 1**). The mean *d*′ score in the Audiovisual Consistent condition was -0.95, which was significantly below chance, *t*(47) = -2.89, *p* = 0.006, Cohen’s *d* = -0.42. The mean *d*′ score in the Audiovisual Inconsistent condition was 1.79 (SD = 2.47), which was significantly above chance, *t*(47) = 4.99, *p* < 0.001, Cohen’s *d* = 0.72. A one-way ANOVA found a significant difference in *d*′ scores across conditions, *F*(2,141) = 16.194, MSE_condition _= 89.93, *p* < 0.001, $\eta_{p}^{2}$ = 0.187. A Bonferroni post-hoc analysis revealed significant pairwise differences between all three display conditions (all *p*’s < 0.05).

### ENDORSEMENT RATE ANALYSIS

**FIGURE 2:**
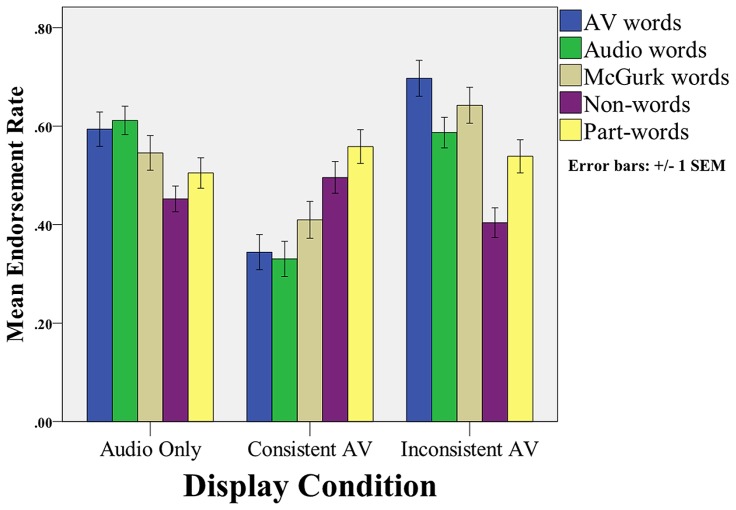
**Mean endorsement rates (i.e., probability of saying “yes”) for the different types of items across conditions**.

We report the endorsement rates for each type of test item in **Figure 2**. We first compared endorsement rates across item type and condition in a 5 (item type) × 3 (display) mixed-factor Repeated Measures ANOVA, where item type was a within-subjects factor and display was between-subjects. In this analysis, there was a significant main effect for item type [*F*(4,564) = 4.53, MSE = 0.21, *p* = 0.001, $\eta_{p}^{2}$ = 0.031], a significant main effect for display condition [*F*(2,141) = 16.68, MSE = 1.42, *p* < 0.001, $\eta_{p}^{2}$ = 0.191], and a significant interaction between item type and display [*F*(8,564) = 11.63, MSE = 0.531, *p* < 0.001, $\eta_{p}^{2}$ = 0.142].

To further examine the interaction between display condition and endorsement rates, we performed separate One-way ANOVAs comparing endorsement rate across display conditions for each of the five item types (see **Figure 2**). There were significant^1^ main effects of condition on endorsement of the three “word” test items: AV words, *F*(2,143) = 26.03, MSE = 1.58, *p* < 0.001, $\eta_{p}^{2}$ = 0.270; Audio words, *F*(2,143) = 23.78, MSE = 1.17, *p* < 0.001, $\eta_{p}^{2}$ = 0.252; McGurk words, *F*(2,143) = 10.32, MSE = 0.66, *p* < 0.001, $\eta_{p}^{2}$ = 0.128. Subsequent linear contrast analyses^2^ on each of the three word items reveal that endorsement rate was significantly greater in the Audiovisual Inconsistent than in Audio-only and Audiovisual Consistent conditions: AV words, *t*(141) = 5.24, *p* < 0.001, Cohen’s *d* = 0.88; Audio words, *t*(141) = 2.97, *p* = 0.004, Cohen’s *d* = 0.50; McGurk words, *t*(141) = 3.70, *p* < 0.001, Cohen’s *d* = 0.62. There were no significant main effects of condition on endorsement of the two foil test items: part-words, *F*(2,143) = 0.67, MSE = 0.04, *p* = 0.512, $\eta_{p}^{2}$ = 0.033; Non-words, *F*(2,143) = 2.42, MSE = 0.10, *p* = 0.093, $\eta_{p}^{2}$ = 0.009. Since the omnibus ANOVAs were not significant, contrast analyses were not conducted for these two item types.

## DISCUSSION

The goal of the present study was to test whether input from multiple modalities could be integrated during statistical learning, utilizing the McGurk effect to manipulate the perceived statistical structure of a speech stream. We presented learners with an artificial language in which word boundaries were not cued by transitional probabilities. The stream was either presented in isolation (audio-only condition) or synchronized with a visual display that either matched the audio stream (Audiovisual Consistent condition) or was discrepant in two word-final syllables (Audiovisual Inconsistent condition), eliciting a McGurk illusion that altered the statistical structure by adding boundary information.

The results of the present study support our predictions that the McGurk illusion in the Audiovisual Inconsistent condition should facilitate participants’ ability to use statistical cues to segment a continuous speech stream. In the Audio-only and Audiovisual conditions, segmentation performance, as measured by *d*′, was not significantly above chance. In contrast, performance in the Audiovisual Inconsistent (i.e., McGurk) condition was above chance and was significantly greater than the Audio-only and Audiovisual Consistent conditions. In addition, the pattern of endorsement rates supports our conclusions from the *d*′ analysis, as we found a significant effect of display condition on endorsement rates. In particular, participants were significantly more likely to endorse the AV word items in the Audiovisual Inconsistent condition. Because these items were consistent across the audio and visual input during familiarization, audiovisual endorsement rate is independent from participants’ perception of the McGurk items. AV word endorsement rate therefore provides a measure of whether the McGurk illusion affected the global statistical structure of the language. Taken together, the *d*′ and endorsement rate analyses demonstrate a significant increase in segmentation performance in the Audiovisual Inconsistent condition, suggesting that learners are capable of audiovisual integration during statistical learning.

It is worth noting that performance in the Audiovisual Consistent condition was significantly lower than the Audio-only Condition, and this appears to be the result of systematically lower endorsement of word items at test. This is a counter-intuitive finding, as our *a priori* hypothesis was that performance would be similar across the Audio-only and Audiovisual Consistent conditions. Nonetheless, the goal of the Audiovisual Consistent condition was to rule out the possibility that any enhancement in performance in the Audiovisual Inconsistent condition was not merely due to the incorporation of a video display (e.g., through increased attention; see Toro et al., 2005). Since learning was significantly greater in the Audiovisual Inconsistent condition than in either the Audio-only or Audiovisual Consistent conditions, we can conclude that this facilitation of learning in the Audiovisual Inconsistent condition was due to the integrated, illusory percept’s enhancement of the transitional probability cues to word boundaries.

To the best of our knowledge, our results provide the first demonstration of multimodal integration during speech segmentation via statistical learning. In the field of statistical learning, as well as in research on language acquisition, there has been growing support for the involvement of multiple sensory modalities in the learning process. For example, several studies have demonstrated a role for vision (e.g., facial movements) in statistical learning (e.g., Sell and Kaschak, 2009; Cunillera et al., 2010; Mitchel and Weiss, 2010; Thiessen, 2010; Van den Bos et al., 2012). However, these studies have not addressed how cross-modal integration may change the input landscape over which statistical learning takes place. Here, we have demonstrated that learners have the capacity to integrate multimodal input during statistical learning, altering the pattern of speech segmentation.

While the results of the present study establish that the integration of audiovisual information can alter statistical learning, our data do not delineate whether the stored representations include either the integrated percept (e.g., /ni/), or the corresponding unimodal percepts (e.g., audio /mi/ and visual /gi/), or perhaps both. According to modality-specific theories of multisensory integration (e.g., Bernstein et al., 2004), multimodal statistical learning would result in the encoding of sensory-specific representations. Alternatively, many common format theories of audiovisual integration (e.g., Fowler, 2004; Summerfield, 1987; Rosenblum, 2005) hold that each unimodal input is transformed^3^ into a singular amodal signal with a “common currency” across sensory modalities. Our data do not distinguish between these mechanisms of multisensory integration, though future work may be able to adapt our paradigm to directly test (e.g., with a two-alternative-forced choice test) the relative availability of unimodal and multimodal representations after familiarization.

The ability to integrate multimodal perceptual input is consistent with a modality-interactive view of statistical learning. Prior research on statistical learning in a multimodal environment has identified modality-specific constraints on statistical learning (Conway and Christiansen, 2005; see also, Conway and Christiansen, 2006, 2009; Emberson et al., 2011). For example, Conway and Christiansen (2005) observed quantitative advantages in auditory domain for extracting temporal regularities relative to the tactile and visual domain. In addition, the authors reported discrepancies in the kind of structure to which learners were sensitive in each modality. Such modality constraints suggest that statistical learning is governed by an array of modality-specific mechanisms (in contrast to, e.g., Kirkham et al., 2002; Thiessen, 2011). The present study, in concert with recent evidence from multimodal statistical learning paradigms, demonstrates a cross-modal effect during statistical learning. Thus, we have suggested (Mitchel and Weiss, 2011; see also Emberson et al., 2011) that while statistical learning may be governed by modality-specific subsystems, these systems are linked within an interactive network. We propose that associations across modalities produce cross-modal effects on learning observed in the current study. This proposal is consistent with modality-specific theories of multisensory integration (see Bernstein et al., 2004), which propose that audiovisual speech perception results in separate, modality-specific representations that become linked upstream in processing. Furthermore, our proposal is consistent with recent neuroimaging work revealing that sensory encoding employs a distributed network of overlapping cortical regions across senses (e.g., Ghazanfar and Schroeder, 2006; Liang et al., 2013; Okada et al., 2013). For example, unimodal auditory input has been shown to elicit a distinct pattern of neural activity in the primary visual cortex, and vice versa (Liang et al., 2013). These findings provide neural evidence of distinct yet associated processing of sensory information across modalities, which is compatible with the view of multisensory statistical learning posited here.

## Conflict of Interest Statement

The authors declare that the research was conducted in the absence of any commercial or financial relationships that could be construed as a potential conflict of interest.

## Footnotes

1 Significance level was adjusted for multiple comparisons. The corrected alpha for five comparisons was 0.01.

2 Contrast weights were -1, -1, 2 for Audio-only, Audiovisual Consistent, and Audiovisual Inconsistent conditions, respectively

3 It should be noted that not all common format theories propose the necessity of transforming multimodal stimuli into a single representation. For example, Gibson’s (1969) invariant detection view proposes that amodal information is directly available in sensory input, and therefore no translation is necessary.

## References

[B1] Bernstein L. E. Auer E. T. Jr. Moore J. K. (2004). “Audiovisual speech binding: convergence or association,” in *Handbook of Multisensory Processing* eds Calvert G. A. Spence C. Stein B. E. (Cambridge, MA: MIT Press) 203–224

[B2] Brancazio L. Miller J. L. (2005). Use of visual information in speech perception: evidence for a visual rate effect both with and without a McGurk effect. *Percept. Psychophys.* 67 759–769 10.3758/BF03193531 16334050

[B3] Conway C. M. Christiansen M. H. (2005). Modality-constrained statistical learning of tactile, visual, and auditory sequences. *J. Exp. Psychol. Learn. Mem. Cogn.* 31 24–39 10.1037/0278-7393.31.1.24 15641902

[B4] Conway C. M. Christiansen M. H. (2006). Statistical learning within and between modalities: pitting abstract against stimulus specific representations. *Psychol. Sci.* 17 905–912 10.1111/j.1467-9280.2006.01801.x 17100792

[B5] Conway C. M. Christiansen M. H. (2009). Seeing and hearing in space and time: effects of modality and presentation rate on implicit statistical learning. *Eur. J. Cogn. Psychol.* 21 561–580 10.1080/0954144080097951

[B6] Cunillera T. Càmara E. Laine M Rodríguez-Fornells A. (2010). Speech segmentation is facilitated by visual cues. *Q. J. Exp. Psychol. (Hove)* 63 260–274 10.1080/17470210902888809 19526435

[B7] Emberson L. L. Conway C. M. Christiansen M. H. (2011). Timing is everything: changes in presentation rate have opposite effects on auditory and visual implicit statistical learning. *Q. J. Exp. Psychol. (Hove)* 64 1021–1040 10.1080/17470218.2010.538972 21347988

[B8] Fiser J. Aslin R. N. (2002). Statistical learning of higher order temporal structure from visual shape-sequences. *J. Exp. Psychol. Learn. Mem. Cogn.* 28 458–467 10.1037/0278-7393.28.3.458 12018498

[B9] Fowler C. A. (2004). “Speech as a supramodal or amodal phenomenon,” in *HandBook of Multisensory Processing* edsCalvert G. A. Spence C. Stein B. E. (Cambridge, MA: MIT Press) 189–202

[B10] Ghazanfar A. A. Schroeder C. E. (2006). Is neocortex essentially multisensory? *Trends Cogn. Sci. (Regul. Ed.)* 10 278–285 10.1016/j.tics.2006.04.008 16713325

[B11] Gibson E. J. (1969). *Principles of Perceptual Learning and Development*. New York: Appleton-Century-Crofts.doi: 10.1126/science.168.3934.958

[B12] Glicksohn A. Cohen A. (2013). The role of cross-modal associations in statistical learning. *Psychon. Bull. Rev.* 1161–1169 10.3758/s13423-013-0458-4 23716019

[B13] Green K. P. (1998). “The use of auditory and visual information during phonetic processing: implications for theories of speech perception,” in *Hearing by Eye II: Advances in the Psychology of Speechreading and Auditory–Visual Speech* edsCampbell R. Dodd B. Burnham D. (Hove: Psychology Press) 3–25

[B14] Kirkham N. Z. Slemmer J. A. Johnson S. P. (2002). Visual statistical learning in infancy: evidence for a domain-general learning mechanism. *Cognition* 83 B35–B42 10.1016/S0010-0277(02)00004-5 11869728

[B15] Liang M. Mouraux A. Hu L. Iannetti G. D. (2013). Primary sensory cortices contain distinguishable spatial patterns of activity for each sense. *Nat. Commun.* 4 1979 10.1038/ncomms2979 PMC3709474 23752667

[B16] MacDonald J. McGurk H. (1978). Visual influences on speech perception processes. *Percept. Psychophys.* 24 253–257 10.3758/BF03206096 704285

[B17] Massaro D. W. (1998). *Perceiving Talking Faces: From Speech Perception to a Behavioral Principle*. Cambridge, MA: MIT Press

[B18] McGurk H. MacDonald J. (1976). Hearing lips and seeing voices. *Nature* 264 746–748 1012311 10.1038/264746a0

[B19] Mitchel A. D. Weiss D. J. (2010). What's in a face? Visual contributions to speech segmentation. *Lang. Cogn. Process.* 25 456–482 10.1080/01690960903209888

[B20] Mitchel A. D. Weiss D. J. (2011). Learning across senses: cross-modal effects in multisensory statistical learning. *J. Exp. Psychol. Learn. Mem. Cogn.* 37 1081–1091 10.1037/a0023700 21574745 PMC4041380

[B21] Mitchel A. D. Weiss D. J. (2013). Visual speech segmentation: using facial cues to locate word boundaries in continuous speech. *Lang. Cogn. Process.* 1–10 10.1080/01690965.2013.791703 [Epub ahead of print]. PMC4091796 25018577

[B22] Okada K. Venezia J. H. Matchin W. Saberi K. Hickok G. (2013). An fMRI study of audiovisual speech perception reveals multisensory interactions in auditory cortex. *PLoS ONE* 8:e68959 10.1371/journal.pone.0068959 PMC3689691 23805332

[B23] Rosenblum L. D. (2005). “Primacy of multimodal speech perception,” in *The HandBook of Speech Perception* edsPisoni D. Remez R. (Malden, MA: Blackwell Publishing) 51–78

[B24] Saffran J. R. Aslin R. N. Newport E. L. (1996). Statistical learning by 8-month-old infants. *Science* 274 1926–1928 10.1126/science.274.5294.1926 8943209

[B25] Saffran J. R. Johnson E. K. Aslin R. N. Newport E. L. (1999). Statistical learning of tone sequences by human infants and adults. *Cognition* 70 27–52 10.1016/S0010-0277(98)00075-4 10193055

[B26] Saffran J. R. Newport E. L. Aslin R. N. (1996). Word segmentation: the role of distributional cues. *J. Mem. Lang.* 35 606–621 10.1006/jmla.1996.0032

[B27] Seitz A. R. Kim R. van Wassenhove V. Shams L. (2007). Simultaneous and independent acquisition of multisensory and unisensory associations. *Perception* 36 1445–1453 10.1068/p5843 18265827

[B28] Sell A. J. Kaschak M. P. (2009). Does visual speech information affect word segmentation? *Mem.Cogn.* 37 889–894 10.3758/MC.37.6.889 PMC4606934 19679867

[B29] Shams L. Seitz A. R. (2008). Benefits of multisensory learning. *Trends Cogn. Sci. (Regul. Ed.)* 12 411–417 10.1016/j.tics.2008.07.006 18805039

[B30] Shimojo S. Shams L. (2001). Sensory modalities are not separate modalities: plasticity and interactions. *Curr. Opin. Neurobiol* 11 505–509 10.1016/S0959-4388(00)00241-5 11502399

[B31] Stein B. E. Stanford T. R. (2008). Multisensory integration: current issues from the perspective of the single neuron. *Nat. Rev. Neurosci.* 9 255–266 10.1038/nrn2331 18354398

[B32] Summerfield A. Q. (1987). “Some preliminaries to a comprehensive account of audio-visual speech perception,” in *Hearing by Eye* edsDodd B. Campbell R. (London: Erlbaum Associates) 3–51

[B33] Thiessen E. D. (2010). Effects of visual information on adults’ and infants’ auditory statistical learning. *Cogni. Sci.* 34 1093–1106 10.1111/j.1551-6709.2010.01118.x 21564244

[B34] Thiessen E. D. (2011). Domain general constraints on statistical learning. *Child Dev.* 82 462–470 10.1111/j.1467-8624.2010.01522.x 21410909

[B35] Toro J. M. Sinnett S. Soto-Faraco S. (2005). Speech segmentation by statistical learning depends on attention. *Cognition* 97 B25–B34 10.1016/j.cognition.2005.01.006 16226557

[B36] Van den Bos E. Christiansen M. H. Misyak J. B. (2012). Statistical learning of probabilistic nonadjacent dependencies by multiple-cue integration. *J. Mem. Lang.* 67 507–520 10.1016/j.jml.2012.07.008

[B37] Weiss D. J. Gerfen C. Mitchel A. D. (2010). Colliding cues in word segmentation: the role of cue strength and general cognitive processes. *Lang. Cogn. Process.* 25 402–422 10.1080/01690960903212254

[B38] Weiss D. J. Gerfen C. Mitchel A. D. (2009). Speech segmentation in a simulated bilingual environment: a challenge for statistical learning? *Lang. Learn. Dev.* 5 30–49 10.1080/15475440802340101 24729760 PMC3981102

